# Evaluation of sodium levels and changes in foods from the top 20 Canadian restaurant chains (2016–2020) against UK National Salt Reduction Maximum targets

**DOI:** 10.1371/journal.pone.0328525

**Published:** 2025-08-12

**Authors:** Caroline G. Middleton, Yahan Yang, Mavra Ahmed, Jennifer J. Lee, Mary R. L’Abbé

**Affiliations:** 1 Department of Nutritional Sciences, Temerty Faculty of Medicine, University of Toronto, Toronto, Ontario, Canada; 2 Joannah & Brian Lawson Centre for Child Nutrition, University of Toronto, University of Toronto, Toronto, Ontario, Canada; 3 School of Nutrition, Toronto Metropolitan University, Toronto, Ontario, Canada.; AIIMS Jodhpur: All India Institute of Medical Sciences - Jodhpur, INDIA

## Abstract

High sodium intake contributes to hypertension, a leading risk factor for cardiovascular disease. Over 50% of Canadians regularly consume prepackaged and restaurant foods, which account for more than 70% of dietary sodium. Canada currently lacks public health strategies to address sodium levels in restaurant menu items, while the UK’s voluntary sodium reduction program (with targets set through the National Salt Reduction Initiative [NSRI]) led to significant reductions in sodium. The objectives were to compare sodium levels in Canadian restaurant menu items in 2020 to the UK NSRI 2024 targets and analyze changes between 2016 and 2020. Data were obtained from the University of Toronto Menu-FLIP (Food Label Information and Price) database, which includes over 20,000 items from 141 Canadian chain restaurants. A total of 3,616 menu items from the top 20 Canadian chains were assessed, of which 1,914 items in categories with UK NSRI 2024 targets were identified and compared to those targets, and 607 items were matched between 2016 and 2020 and analyzed for sodium changes. More than half (56.6%, *n* = 1,083/1,914) of items exceeded UK NSRI targets. Sodium (mg/100 g) showed a large decrease in 39.5% (*n* = 100/607) of items, a medium decrease in 15.8% (*n* = 63/607), little change in 28.9% (*n* = 182/607), a medium increase in 5.3% (*n* = 68/607), and a large increase in 10.5% (*n* = 194/607) from 2016 to 2020. The prevalence and magnitude of sodium changes varied by food category. Overall, there was a statistically significant but nutritionally insignificant reduction in sodium per serving from 2016 to 2020 (−24 ± 819 mg, *p* < 0.01). Canadian restaurant menu items were high in sodium, with more than half surpassing the UK NSRI targets. The observed increases and decreases in sodium highlight the need for Health Canada to set and for industry to adopt sodium reduction targets for restaurant menu items, similar to those in the UK.

## Introduction

High sodium intake has been associated with an elevated risk of noncommunicable diseases (NCDs) [1,2], including increased vulnerability to hypertension and adverse cardiovascular events such as stroke and heart disease [3,4]. Notably, hypertension remains the primary modifiable risk factor for global mortality [5] and affects nearly one in four Canadian adults [4]. This underscores the need for reducing sodium intake across all age groups, as Canadians, on average, consume 2,760 mg/day of sodium [6], exceeding the recommended threshold for Chronic Disease Risk Reduction (CDRR) of 2,300 mg/day [7].

Reducing the sodium content of restaurant foods is pivotal to curbing excessive sodium intake and alleviating the burden of NCDs. Approximately 70% of sodium consumed by Canadians comes from prepackaged foods and foods prepared outside the home, including restaurants and other foodservice establishments (e.g., school or hospital cafeterias, catering) [8]. With over half the population routinely eating out at fast-food and sit-down restaurants [9], several US studies have demonstrated a strong correlation between restaurant food consumption and excessive intakes of energy, saturated fat, sodium, and sugars [10–13]. However, the absence of comprehensive menu labelling regulations in Canada has limited consumers’ access to sodium information for restaurant menu items, hindering their ability to make well-informed and health-conscious dietary choices when eating out.

Canada aims to achieve a 30% reduction in mean population sodium intake by 2025, aligned with the World Health Organization’s global targets for preventing NCDs [14]. Although the 2010 *Sodium Reduction Strategy for Canada* included a variety of recommendations concerning population-level sodium intake, research, education, and monitoring [15], Health Canada has never set specific targets for restaurant foods. Current efforts rely on voluntary measures that establish targets for sodium reduction in prepackaged foods. Voluntary targets to lower sodium in prepackaged foods have yielded minimal reductions, underscoring the necessity for more effective interventions [16,17]. By 2017, only 14% of food categories had met Health Canada’s 2016 voluntary sodium reduction targets [16]. In contrast, the UK’s voluntary sodium reduction initiative has substantially cut sodium in prepackaged foods, resulting in a 16% drop in population sodium intake and a 17% reduction in sodium density due to a 12.0 mg/100 g reduction through reformulation [18]. Notably, this initiative has continued to evolve by revising sodium reduction targets for prepackaged foods and introducing new targets for restaurant foods in September 2020, with expected achievement by 2024. Established through the UK National Salt Reduction Initiative (NSRI), these new sodium reduction targets include ten key food categories that contribute most to the UK population’s sodium intake benchmarks identified from the most popular dishes sold in UK restaurants and an additional food category established for children’s meals [19]. This initiative aims to further reduce sodium consumption from restaurant menu items and promote food reformulation [20], presenting a robust framework for sodium reduction that Canada could adopt [21]. Given the prevalence of meals consumed outside the home, analyzing sodium levels in Canadian restaurant menu items is crucial to monitoring progress in the industry and foodservice sector regarding sodium reduction. This analysis involves comparing the sodium levels in Canadian restaurant menu items to the voluntary UK NSRI targets, which have proven effective in reducing sodium levels.

Therefore, the objectives of the present study were to 1) assess sodium levels in Canadian restaurant menu items in 2020 compared to the UK NSRI targets, and 2) examine sodium changes in matched restaurant menu items between 2016 and 2020 to evaluate whether the restaurant industry has made progress toward reducing sodium content in menu offerings.

## Materials and methods

### Study design

Using the most up-to-date University of Toronto Menu-Food Label Information and Price (Menu-FLIP) database 2020, the sodium levels of Canadian restaurant menu items were examined. The top 20 national chain restaurants in Canada with ≥20 locations were included, representing ~60% of Canada’s chain foodservice brand share (2016–2019), based on Canada sales [22]. S1 Table shows the list of restaurant chains included in the analysis. To examine the changes in the sodium levels of reformulated items, a longitudinal study was conducted on the nutrition information of matched restaurant menu items from Menu-FLIP 2016 and 2020, as was done earlier [23].

### Menu-FLIP database

Data on Canadian chain restaurant menu items for this study were obtained from the University of Toronto Menu-FLIP (Food Labelling Information and Price) database, which was established in 2010 for collecting the nutrition information of Canadian chain restaurant foods with 20 or more outlets across Canada. Detailed methodology has been published elsewhere [23]. Briefly, Menu-FLIP 2020 contains over 20,000 menu items from 141 Canadian chain restaurants, representing over 70% of the market share of food service establishments in Canada [23]. Vendors that are more informal but corporately affiliated, such as food trucks or carts (e.g., BeaverTails, poutine trucks), were not included. Information collected included identifiers, serving size, energy, and 13 core nutrients as listed on the current Nutrition Facts table for prepackaged foods, if available [24]. Menu items were categorized by restaurant type, based on the presence or absence of table service (e.g., sit-down restaurants offer table service, while fast food or takeaway do not), and by restaurant chain (e.g., McDonald’s, Subway). Menu items were categorized by menu placement (e.g., entrée, side, dessert) and further subcategorized by food type (e.g., burgers, pizzas, fries). Validation of nutritional information (e.g., Atwater calculations for all menu items, which estimate the energy contribution from protein, fat, and carbohydrates) and categories was completed by three reviewers. Duplicate menu items of the same size and items with missing or implausible nutritional information, as identified through data validation (e.g., results from Atwater calculation did not agree with the reported energy, > 20% difference), were excluded to increase the accuracy of data.

Data collected in Menu-FLIP 2016 contained 12,215 restaurant menu items from 96 chains, using the same categorization process described above for 2020. Detailed methodology of Menu-FLIP 2016 can be found elsewhere [23].

### UK NSRI targets

Public Health England has published its fifth set of voluntary sodium reduction targets for all foods, aiming to achieve them by 2024, alongside calorie reduction objectives [19]. The latest version of sodium reduction targets (mg/100 g) for prepackaged foods includes 84 specific food groups (76 from 2017 and 8 new) that contribute the most to the UK population’s sodium intake [19]. The Department of Health has established additional sodium reduction targets for 11 food categories (24 subcategories) based on the ten most popular dishes sold in UK restaurants and a specific target for children’s meals [19]. These targets build upon the success of the UK’s earlier salt reduction campaign, recognized as one of the most effective strategies for reducing sodium at the population level [18]. This campaign has served as a model for similar initiatives in other countries, such as Canada, which based its sodium industry targets on those from the UK, although Canada has yet to publish any targets for restaurant foods.

This study used the maximum sodium targets outlined in Public Health England’s *Eating Out, Takeaway and Delivery Sector Maximum per Serving Salt Targets* [19]. These targets were specifically designed for restaurant menu items and were used to compare sodium levels in restaurant menu items in categories with UK NSRI targets for 2024 [19]. For items without a specific restaurant UK NSRI target, sodium targets for prepackaged foods outlined in Public Health England’s *Salt Reduction Targets for 2024* were used, as retailers and manufacturers are also expected to ensure their foods adhere to these targets [19].

### Comparison of sodium levels in Canadian restaurant menu items in 2020 to UK NSRI targets

The top 20 Canadian restaurant chains were identified based on Canadian market share using the Consumer Foodservice in Canada report from Euromonitor [22]. Restaurant menu items from the top 20 Canadian restaurant chains were assessed (*n* = 3,616). Beverages, ice cream, and other miscellaneous products (e.g., sauces and condiments) (*n* = 1,702) were excluded since they typically do not contain a significant quantity of sodium or were not advertised by restaurants as foods for sale. The final analytic sample size from menu-FLIP 2020 included 1,914 unique restaurant menu items. Restaurant menu items were categorized into UK NSRI food categories, including two sets of targets (11 categories for restaurant foods and 84 categories for prepackaged foods), using the category name and description per the sodium reduction targets 2024 [19]. These items were first evaluated against the *Eating Out, Takeaway, and Delivery Sector Maximum per Serving Salt Targets* categories for restaurant foods [19]. If restaurant targets were not applicable, the *Salt Reduction Targets for 2024* categories for prepackaged foods were applied as recommended [19]. Of the total 95 UK NSRI food categories, 20 (8 categories for restaurant foods and 12 categories for prepackaged foods) were used for our sample. Sodium levels of items were then compared against their respective maximum UK NSRI target (mg/100 g) for 2024. Each menu item underwent a binary classification, either complying with or exceeding thresholds.

### Changes in sodium levels of matched menu items between 2016 and 2020

For our longitudinal analyses, restaurant menu items with UK NSRI targets for 2024 were manually matched between 2016 and 2020 to monitor changes in sodium content over time. Exact matches, defined by identical product names, were identified by comparing product names in the databases, and the corresponding “Product ID” was recorded. Close matches were further examined using the product description within the “Food item” variable to ensure plausibility. Due to few restaurants providing data at the time of the Menu-FLIP 2016 database collection, ten restaurants from 2020 were excluded from the longitudinal study (*n* = 1,407), resulting in a final sample of 607 matched restaurant menu items between 2016 and 2020. Sodium changes were calculated as the percent difference between sodium levels in 2020 and 2016. These changes were categorized as follows: a “large decrease” was defined as a reduction of ≥−15%, a “medium decrease” was defined as a reduction of −5% to −14.9%, and “little change” was defined as a change of less than ±4.9%. A “medium increase” was defined as an increase of +5% to +14.9%, while a “large increase” represented a rise of ≥+15% in sodium content. These thresholds are aligned with the thresholds established for “a little” and “a lot” in the Canadian nutrition labelling regulations [24]. In addition, matched items were analyzed to compare changes in sodium (mg/100 g), calories (kcal/100 g), and serving size (g) over time. These comparisons aimed to offer further insights into potential reasons for the observed changes in sodium content, as previous research has shown that adjusting calories and serving sizes are common strategies used by industry to influence sodium levels per serving [25,26].

### Statistical analysis

Analyses were performed using R Studio (version 4.0.2, RStudio, Boston, MA, USA). Medians and interquartile range (IQR) were calculated by UK NSRI food category. Sodium levels for restaurant menu items (*n* = 1,914) in 2020 were compared against their respective UK NSRI targets. The proportion of food categories exceeding NSRI targets was reported by UK NSRI food category and subcategory. For longitudinal analyses (*n* = 607), sodium changes were calculated as the percent difference between 2016 and 2020. Descriptive statistics and pairwise t-tests were used to compare sodium per kcal/100 g and per serving, with p-values <0.05 considered significant.

## Results

### Comparison of sodium levels in Canadian restaurant menu items in 2020 to UK NSRI targets

**Fig 1:**
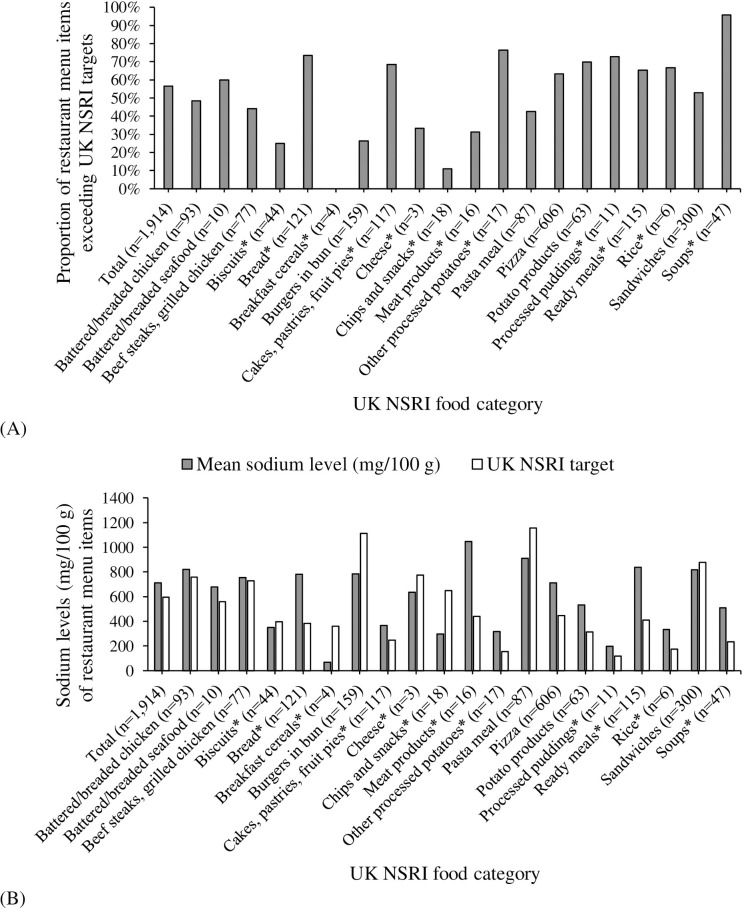
Sodium levels in Canadian restaurant menu items compared to UK National Salt Reduction Initiative (NSRI) targets by UK NSRI food category. (A) Proportion (%) of restaurant items with sodium levels exceeding UK NSRI targets by UK NSRI food category. (B) Mean sodium levels (mg/100 g) in restaurant menu items compared to UK NSRI targets by UK NSRI food category. * UK NSRI food category for prepackaged foods was used because no category for restaurant foods was set.

Of the 1,914 restaurant foods, 56.6% (*n* = 1,083) exceeded UK NSRI targets. Fig 1A shows the proportion of restaurant menu items that exceeded the UK NSRI targets by UK NSRI food category. Food categories with the highest proportion of items (for categories with ≥20 items) exceeding UK NSRI targets were *soups* (95.7%, *n* = 45/47), *bread* (73.6%, *n* = 89/121), and *potato products* (69.8%, *n* = 44/63). *Cakes, pastries, fruit pies* (68.4%, *n* = 80/117), *ready meals* (including prepared meals, meal kits, salads, and more) (65.2%, *n* = 75/115), and *pizza* (63.2%, n = 383/606) are also alarmingly high (>60%). Food subcategories with the highest proportion of items (for subcategories with ≥20 items) exceeding UK NSRI targets include *yeast-raised morning goods* (e.g., bagels) (96.0%, *n* = 48/50), *bread and rolls with additions* (e.g., cheese bread) (88.0%, *n* = 22/25), and *seasoned fries* (81.8%, *n* = 36/44) (S2 Table). Fig 1B shows the mean sodium levels (mg/100 g) in restaurant menu items compared to UK NSRI targets, highlighting higher sodium content in several food categories. *Meat products* showed the highest sodium levels (mean ± SD = 1,047 ± 1,905 mg/100 g), followed by *pasta meals* (910 ± 922), *ready meals* (838 ± 936), *battered/breaded chicken* (822 ± 647), and *sandwiches* (818 ± 883). Several food categories had average sodium levels (mg/100 g) exceeding UK NSRI targets, including *breads* (782 vs. 384), *meat products* (1,047 vs. 441), and *ready meals* (838 vs. 410). In contrast, *chips and snacks* (298 vs. 650), *pasta meals* (910 vs. 1,157), and *burgers in buns* (786 vs. 1,111) had averages below their UK NSRI targets (S2 Table).

### Changes in sodium levels of matched menu items between 2016 and 2020

**Fig 2:**
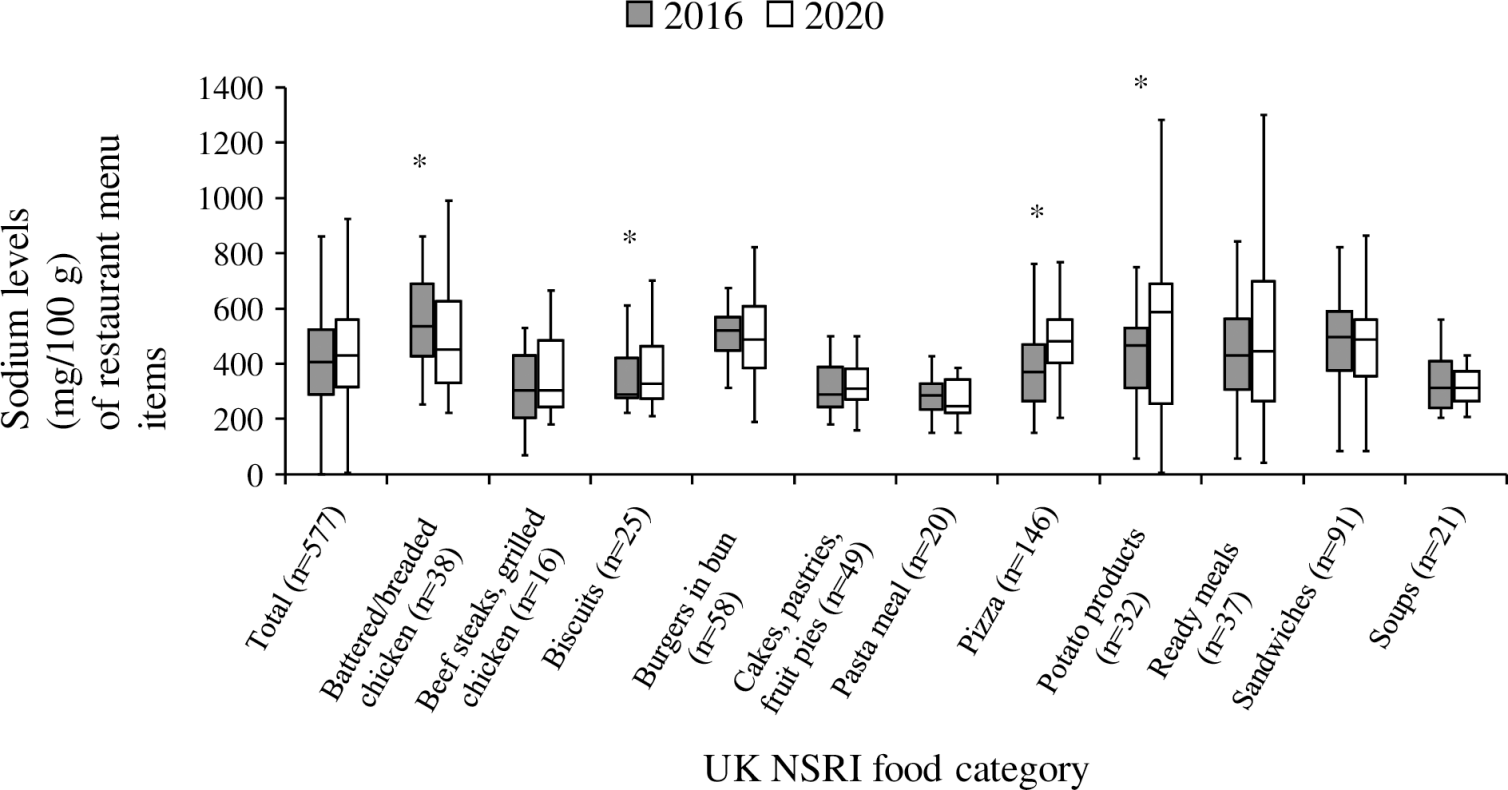
Sodium levels (mg/100 g) in matched restaurant menu items in 2016 and 2020 by UK NSRI food categories with ≥ 10 items. Matched restaurant menu items from the top 20 Canadian chains in the Menu-FLIP database were examined to compare sodium levels between 2016 and 2020. These items were classified according to the UK NSRI food categories [19]. Categories with fewer than 10 menu items were excluded from the figure. Comparisons were made using the Wilcoxon signed-rank test to identify any significant differences between the years. The boxplot represents the interquartile range, with the median indicated by a horizontal line inside the box. *p < 0.05.

Table 1 summarizes the sodium changes in matched restaurant menu items between 2016 and 2020, organized by UK NSRI food categories. Overall, 39.5% (*n* = 100/607) of items showed a large decrease in sodium levels (mg per serving), while 15.8% (*n* = 63/607) experienced a medium decrease, 28.9% (*n* = 182/607) saw little change, 5.3% (*n* = 68/607) had a medium increase, and 10.5% (*n* = 194/607) showed a large increase. On average, sodium levels decreased by −203 ± 732 mg per serving (large decrease) (mean ± SD), −78 ± 744 mg (medium decrease), and −27 ± 859 mg (little change), while they increased by 21 ± 720 mg (medium increase) and 62 ± 884 mg (large increase) per serving (Table 2). Despite the variability, there was an overall small but significant reduction in sodium levels per serving (−24 ± 819 mg, *p* < 0.01); however, there was also a large overall increase in sodium per 100 g (50 ± 307 mg, *p* < 0.01). Items with reduced sodium also exhibited a significant increase in serving size and decreases in sodium and calorie content per 100 g (*p* < 0.01). Conversely, items with increased sodium showed reductions in serving size but increases in sodium and calorie content per 100 g (*p* < 0.01). Sodium changes differed across food categories, with significant reductions observed only in *battered/breaded chicken*, while *pizza*, *potato products*, and *biscuits* all showed significant increases in sodium levels per mg/100 g (Fig 2).

**Table 1:** Sodium level changes in matched restaurant menu items (2016–2020) by UK NSRI food category.

UK NSRI Food Category	*n*	Sodium Changes in Restaurant Menu Items (*n*, %)				
		Large decrease	Medium decrease	Little change	Medium increase	Large increase
Total	**607**	**100 (39.5)**	**63 (15.8)**	**182 (28.9)**	**68 (5.3)**	**194 (10.5)**
Battered/breaded chicken	38	15 (0.0)	2 (100.0)	11 (0.0)	4 (0.0)	6 (0.0)
Battered/breaded seafood	1	0 (6.3)	0 (37.5)	0 (12.5)	0 (12.5)	1 (31.3)
Beef steaks, grilled chicken	16	1 (8.0)	2 (12.0)	2 (44.0)	5 (20.0)	6 (16.0)
Biscuits*	25	2 (18.2)	5 (25.0)	11 (36.4)	4 (9.1)	3 (11.4)
Bread*	44	8 (0.0)	4 (0.0)	16 (100.0)	5 (0.0)	11 (0.0)
Breakfast cereals*	4	0 (25.9)	0 (13.8)	4 (27.6)	0 (17.2)	0 (15.5)
Burgers in bun	58	15 (12.2)	10 (16.3)	16 (46.9)	9 (16.3)	8 (8.2)
Cakes, pastries, fruit pies*	49	6 (0.0)	8 (0.0)	23 (100.0)	4 (0.0)	8 (0.0)
Chips and snacks*	1	0 (33.3)	0 (0.0)	1 (66.7)	0 (0.0)	0 (0.0)
Meat products*	3	1 (0.0)	0 (11.1)	2 (88.9)	0 (0.0)	0 (0.0)
Other processed potatoes*	9	0 (35.0)	0 (30.0)	8 (25.0)	0 (10.0)	1 (0.0)
Pasta meal	20	7 (5.5)	2 (65.1)	5 (12.3)	0 (2.7)	6 (14.4)
Pizza	146	8 (15.6)	4 (56.3)	18 (25.0)	21 (3.1)	95 (0.0)
Potato products	32	5 (22.2)	1 (33.3)	8 (44.4)	0 (0.0)	18 (0.0)
Processed puddings*	9	2 (10.8)	0 (32.4)	4 (24.3)	0 (13.5)	3 (18.9)
Ready meals*	37	4 (33.3)	5 (66.7)	9 (0.0)	7 (0.0)	12 (0.0)
Rice*	3	1 (24.2)	0 (13.2)	0 (34.1)	0 (20.9)	2 (7.7)
Sandwiches	91	22 (14.3)	19 (9.5)	31 (61.9)	7 (4.8)	12 (9.5)
Soups*	21	3 (16.5)	1 (32.0)	13 (30.0)	2 (10.4)	2 (11.2)

*n* = 607. Values are n (%), organized alphabetically based on UK NSRI food categories [19]. Sodium changes were calculated as the percent difference between sodium levels in 2020 and 2016. Sodium changes were defined as follows: a “large decrease” refers to a reduction of ≥−15% in sodium content, a “medium decrease” indicates a reduction of −5% to −14.9%, and a “little change” reflects a change of less than ±4.9%, as defined in the Canadian nutrition labelling regulations [24]. A “medium increase” represents an increase of +5% to +14.9%, while a “large increase” signifies a rise of ≥+15% in sodium content. *UK NSRI food categories for prepackaged foods were used because no category for restaurant foods was set.

**Table 2:** Changes in sodium levels, serving sizes, and calories in matched restaurant menu items (2016–2020) by sodium reduction or increase.

Sodium Changes in Restaurant Menu Items	*n* (%)	Sodium levels (mg/serving), Mean ± SD			Sodium (mg/100 g), Mean ± SD			Serving size (g), Mean ± SD			Calories (kcal/100 g), Mean ± SD		
		2016	2020	Avg change	2016	2020	Avg change	2016	2020	Avg change	2016	2020	Avg change
Total	**607 (100)**	**774 ± 549**	750 ± 608*	**−24 ± 819**	**414 ± 171**	464 ± 255**	**50 ± 307**	**162 ± 157**	159 ± 149**	**−3 ± 216**	**229 ± 104**	278 ± 168**	**49 ± 198**
Large decrease	100 (17)	856** ± **559	653** ± **472	−203 **± **732	518** ± **174	350** ± **149	−168 ± 229	173 ± 122	191 ± 135	18 ± 182	398 ± 234	383 ± 232	−15 ± 330
Medium decrease	63 (10)	845** ± **489	767** ± **561	−78 **± **744	474 **± **145	431** ± **134	−43 ± 197	180 **± **105	190 **± **123	10 ± 162	407 ± 219	426 ± 239	19 ± 324
Little change	182 (30)	740 ± 614	713 ± 601	−27 **± **859	394 ± 159	394 ± 157	0 ± 223	196 ± 155	193 ± 157	−3 ± 221	407 ± 317	413 ± 322	6 ± 452
Medium increase	68 (11)	746** ± **451	767** ± **561	21 **± **720	433** ± **199	474** ± **215	41 ± 293	194 ± 132	178 ± 134	−16 ± 188	412 ± 240	426 ± 239	14 ± 339
Large increase	194 (32)	751** ± **525	813 **± **711	62 ± 884	353** ± **144	597 **± **344	244 ± 373	221 ± 135	148 ± 131	−73 ± 188	378 ± 291	424 ± 328	46 ± 438

*n* = 607. Sodium changes were defined as follows: a “large decrease” refers to a reduction of ≥−15% in sodium content, a “medium decrease” indicates a reduction of −5% to −14.9%, and a “little change” reflects a change of less than ±4.9%, as defined in the Canadian nutrition labelling regulations [24]. A “medium increase” represents an increase of +5% to +14.9%, while a “large increase” signifies a rise of ≥+15% in sodium content. Abbreviations: SD, Standard deviation; Avg, Average. *p < 0.05. **Paired *t*-*t*ests, *p* < 0.01.

## Discussion

This study provides a comprehensive comparison of sodium levels in a wide array of menu items from Canadian sit-down and fast-food restaurants. This data can serve as a benchmark for assessing progress in the sector, particularly in comparison to the UK NSRI targets, which have effectively reduced sodium levels in restaurant menu items. The findings reveal alarmingly high average sodium levels in Canadian restaurant menu items, with approximately 56% of items exceeding the recommended UK NSRI targets. Notably, many individual restaurant items (often not comprising a full meal, such as a burger without accompanying fries) surpassed the 2024 sodium reduction targets set by the UK NSRI. Given the significant proportion of meals consumed outside the home [8], coupled with the high rates of hypertension and cardiovascular events [3,4], the results of this study demonstrate the need for increased efforts to reduce sodium in the restaurant and foodservice sectors, as these areas have been largely overlooked in Canadian policy but are necessary for addressing excessive sodium consumption.

Our results highlight the current high and variable sodium levels in Canadian restaurant foods, aligning with similar findings in Canada and the US [25–29]. Particularly concerning is that over 50% of the assessed menu items surpass the UK NSRI targets, with *soups* (95.7%, *n* = 45/47), *bread* (73.6%, *n* = 89/121), and *potato products* (69.8%, *n* = 44/63) being the most problematic categories encompassing many products. *Meat products*, *pasta meals*, and *ready meals (including prepared meals, meal kits, salads, and more)* also raise concern, consistent with previous US findings [28]. The large variation in sodium levels within these categories indicates that reducing sodium is both achievable and palatable in terms of taste preferences [30]. Interestingly, *breakfast cereals* and some *chips and snacks* were the only categories where sodium levels (mg/100 g) were below UK NSRI targets. Yet, many foods in these categories, such as sugar-frosted cornflakes and potato chips, are high in sugar, saturated fat, and/or calories, which should be considered when interpreting these results. Comprehensive and specific benchmarks for different food categories are needed to support sodium reduction across the Canadian restaurant and foodservice sectors.

Moreover, our results indicate an overall statistically significant yet nutritionally insignificant decrease in sodium per serving in Canadian restaurant menu items between 2016 and 2020, with a reduction of –24 (SD ± 819) mg/serving, despite increases in some categories. This study shows that current industry efforts to decrease sodium levels in restaurant items in Canada have produced mostly inconsistent and negligible results, which highlights a concerning lack of oversight of the restaurant sector. Addressing this issue is critical for improving sodium intake in the Canadian population. Often, reductions were driven by smaller portion sizes rather than product reformulation, consistent with prior research on “shrinkflation” in Canadian foods [25]. Because serving size reductions can mask sodium density, our analysis also examined sodium per 100 g, providing a more accurate picture of reformulation. These findings underscore the importance of setting sodium targets per serving and per 100 g, alongside implementing clear guidelines for menu labeling and transparency [31].

Current efforts to reduce sodium levels in Canadian restaurants remain insufficient. Although voluntary sodium reduction targets have been established for prepackaged foods in Canada since 2012 [32] and for restaurant menu items in the US [33] and the UK [19], Canada has yet to implement specific targets for sodium reduction in restaurant menu items. Compared to countries like the UK, which has a government-industry agreement to lower sodium levels [19], restaurant items in Canada consistently have higher sodium content. For example, McDonald’s Chicken McNuggets contain 600 mg of sodium per 100 g serving in Canada, whereas similar-sized servings in the UK contain only 240 mg, nearly two-and-a-half times less [30]. Moreover, there has been no noticeable reduction in the proportion of Canadian restaurant menu items exceeding sodium’s CDRR of 2,300 mg/day [4].

Achieving meaningful sodium reduction in restaurants will require the establishment of strict targets and more rigorous monitoring. Research has shown that reformulating menu items to lower sodium does not compromise consumer acceptance, supporting the feasibility of such changes [34,35]. Several countries have already made progress in this area, many of which include restaurant and out-of-home foods as a priority area [36]. For example, the United States introduced voluntary sodium targets in 2021 with the aim of reducing average population intake by 12% by 2025, which includes sodium targets for some restaurant foods [37]. Countries such as Chile, France, and Italy have prioritized reformulation of high-contributing sources such as bread, while jurisdictions including South Africa and Argentina have adopted mandatory, legally enforceable sodium limits for key food categories [36]. Canada has yet to establish sodium reduction targets for the restaurant sector. To address this gap, Canada should implement a phased, category-specific framework, beginning with the most significant dietary sources of sodium, such as breads and processed meats [36]. A mandatory approach would necessitate explicit sodium thresholds, defined timelines, and federal oversight. In the context of voluntary implementation, robust and independent monitoring of sodium levels in restaurant and processed foods is essential to ensure transparency, industry accountability, and sustained progress [36]. Aligning national efforts with established international models would strengthen the public health impact of sodium reduction policies and reinforce Canada’s contribution to global noncommunicable disease prevention.

Complementary consumer-directed strategies are also warranted in parallel with regulatory reform. Nutrient menu labelling represents a key avenue for intervention, as evidence suggests that when nutritional information is presented at the point of purchase, a significant proportion of consumers, ranging from 50% to 70%, engage with this information [38]. Canada’s forthcoming front-of-package labelling regulations for prepackaged foods, set to take effect in January 2026, provide a model that could be extended to restaurant menu items [39]. Internationally, voluntary labeling systems such as Nutri-Score have already been applied to restaurant foods in parts of Europe [40], while several jurisdictions in the United States, including New York City and Philadelphia, have implemented mandatory warning labels for high-sodium items [41,42]. In addition to labelling, public health education and food literacy campaigns can play an essential role in shifting consumer preferences and increasing demand for healthier options [43,44]. Campaigns disseminated through social media, television, and school-based initiatives may be particularly effective in raising awareness of sodium content in commonly consumed restaurant items and highlighting the lack of progress in industry reformulation [44]. Taken together, a comprehensive strategy that integrates regulatory policy with consumer education and engagement is likely to yield the most substantial and sustained reductions in population sodium intake [43–45].

One of the main limitations of the study is that it only included data for the top 20 restaurant chains (~60% of Canada’s chain foodservice brand share) [22]; therefore, our sample may not be representative of the entire restaurant food supply because our study examined sodium levels in the largest chain restaurants and did not include smaller chains and independent establishments. Furthermore, the findings presented in this study did not include all menu items from the restaurants represented in the sample, as some matches could not be identified when reformulated foods were reintroduced or rebranded. This limitation may have obscured potentially lower sodium levels in new menu items. Another limitation is that the Canadian category means were not market-share weighted, unlike the UK. The impact of this on our results is uncertain, as it is unclear whether this approach produced inflated or conservative results. A study on the sodium content of processed foods in the UK found that the purchase-weighted mean sodium was 18–35% higher than the unweighted mean sodium levels [46]. Further research is needed to determine how market share influences these restaurant results. Finally, even though our data was collected in 2016 and 2020 and compared to 2024 targets, it is unlikely that there have been major decreases in sodium levels over the past four years, as Canada has not yet established targets or implemented a reduction strategy for the restaurant sector.

## Conclusions

More than half of the items in Canadian restaurants exceed the UK NSRI targets, demonstrating the need for a Canadian sodium reduction strategy that emphasizes reductions in restaurant menu items, similar to those in the UK and the US. From 2016 to 2020, the observed increases and decreases in sodium show that current industry efforts to decrease sodium levels in Canadian restaurant menu items have produced mostly inconsistent and negligible impacts. Although the lower levels observed in restaurant menu items show that sodium reduction is possible, the simultaneous increases seen in some foods demonstrate the need for targets and timelines for sodium reduction in restaurants. Because of the prevalence of eating out, as well as the high rates of hypertension and cardiovascular events, addressing the exceedingly high sodium levels in restaurant items is essential in order to decrease the burden of chronic disease in Canada.

## Supporting information

S1 Table Restaurant menu items from the top 20 chain restaurants in Canada. *n = *1,914. for unique restaurant menu items. *n* = 607 for matched restaurant menu items (2016–2020). Values are *n* (%). Data was retrieved from the top 20 chain restaurants, representing 60% of Canada’s chain foodservice brand share (2016–2019), based on global brand names from Euromonitor International [1].
(DOCX)

S2 Table Proportion of Canadian restaurant menu items that exceed the UK National Salt Reduction Initiative (NSRI) sodium reduction targets by UK NSRI food category and subcategory. *n = *1,914. Values are *n* (%), organized in alphabetical order according to food categories that exceed the UK National Salt Reduction Initiative (NSRI) targets [19]. Data is categorized by UK NSRI food category and subcategory. (*) Indicates UK NSRI food category for prepackaged foods was used because no category for restaurant foods was set.
(DOCX)
