# Supplementary material for: Evaluation of sodium levels and changes in foods from the top 20 Canadian restaurant chains (2016–2020) against UK National Salt Reduction Maximum targets
Source: PLoS One. 2025 Aug 12;20(8):e0328525. doi: 10.1371/journal.pone.0328525 (PMC12342300; doi:10.1371/journal.pone.0328525)
Supplement: S1 Table — n = 1,914. for unique restaurant menu items. n = 607 for matched restaurant menu items (2016–2020). Values are n (%). Data was retrieved from the top 20 chain restaurants, representing 60% of Canada’s chain foodservice brand share (2016–2019), based on global brand names from Euromonitor International [1]. (DOCX) [file pone.0328525.s001.docx]

**S1 Table.** **Restaurant menu items from the top 20 chain restaurants in Canada.**

| Restaurants | | Unique restaurant foods  (*n*, %) | Matched restaurant foods  (*n*, %) |
| --- | --- | --- | --- |
| 1 | A&W | 41 (2.1) | 9 (1.5) |
| 2 | Boston Pizza | 153 (8.0) | 23 (3.8) |
| 3 | Burger King | 68 (3.6) | 32 (5.3) |
| 4 | Dairy Queen | 34 (1.8) | 22 (3.6) |
| 5 | East Side Mario’s | 104 (5.4) | 37 (6.1) |
| 6 | Harvey’s | 46 (2.4) | 23 (3.8) |
| 7 | Jack Astor’s | 46 (2.4) | 9 (1.5) |
| 8 | KFC | 63 (3.3) | 36 (5.9) |
| 9 | Little Caesar’s | 43 (2.3) | 4 (0.7) |
| 10 | McDonald’s | 58 (3.0) | 32 (5.3) |
| 11 | Milestones | 48 (2.5) | 21 (3.5) |
| 12 | Montana’s | 54 (2.8) | 24 (3.9) |
| 13 | Pizza Hut | 377 (19.7) | 115 (18.9) |
| 14 | Pizza Pizza | 157 (8.2) | 21 (3.4) |
| 15 | Starbucks | 70 (3.7) | 19 (3.1) |
| 16 | Subway | 144 (7.5) | 54 (8.9) |
| 17 | Swiss Chalet | 67 (3.5) | 15 (2.5) |
| 18 | The Keg | 74 (3.9) | 30 (4.9) |
| 19 | Tim Horton’s | 163 (8.5) | 68 (11.2) |
| 20 | Wendy’s | 104 (5.4) | 13 (2.1) |
| Total | | **1,914 (100)** | **607 (100)** |

*n=*1,914. for unique restaurant menu items. *n*=607 for matched restaurant menu items (2016–2020). Values are *n* (%). Data were retrieved from the top 20 chain restaurants, representing ~60% of Canada’s chain foodservice brand share (2016–2019) based on Canada sales [1] that are in categories with UK NSRI targets.
