# Supplementary material for: Evaluation of sodium levels and changes in foods from the top 20 Canadian restaurant chains (2016–2020) against UK National Salt Reduction Maximum targets
Source: PLoS One. 2025 Aug 12;20(8):e0328525. doi: 10.1371/journal.pone.0328525 (PMC12342300; doi:10.1371/journal.pone.0328525)
Supplement: S2 Table — n = 1,914. Values are n (%), organized in alphabetical order according to food categories that exceed the UK National Salt Reduction Initiative (NSRI) targets [19]. Data is categorized by UK NSRI food category and subcategory. (*) Indicates UK NSRI food category for prepackaged foods was used because no category for restaurant foods was set. (DOCX) [file pone.0328525.s002.docx]

**S2 Table. Proportion of Canadian restaurant menu items that exceed the UK National Salt Reduction Initiative (NSRI) targets by UK NSRI food category and subcategory.**

| UK NSRI Food Categories | *n* | UK NSRI target  (mg/100 g) | Mean sodium (mg/100 g) | Exceed UK NSRI target, *n (*%) |
| --- | --- | --- | --- | --- |
| Battered or breaded chicken portions and pieces | **93** | **756** | **822** | **45 (48.1)** |
| 200-400kcal | 33 | 663 | 506 | 10 (30.3) |
| Over 400kcal | 51 | 879 | 1,112 | 33 (64.7) |
| Under 200kcal | 9 | 404 | 335 | 2 (22.2) |
| Battered or breaded seafood-based meals * | **10** | **560** | **678** | **6 (60.0)** |
| Bitesize seafood meals | 6 | 661 | 741 | 3 (50.0) |
| Fish fillet meals | 4 | 408 | 584 | 3 (75.0) |
| Beef steaks, grilled chicken and roast main meals | **77** | **729** | **753** | **34 (44.2)** |
| Beef steaks, grilled chicken and roast main  meals | 77 | 729 | 753 | 34 (44.2) |
| Biscuits * | **44** | **397** | **349** | **11 (25.0)** |
| Savoury biscuits | 7 | 700 | 559 | 1 (14.3) |
| Sweet biscuits | 37 | 340 | 309 | 10 (27.0) |
| Bread * | **121** | **384** | **782** | **89 (73.6)** |
| Bread and rolls | 37 | 405 | 472 | 18 (48.7) |
| Bread and rolls with additions | 25 | 430 | 916 | 22 (88.0) |
| Morning goods - powder raised | 9 | 475 | 257 | 1 (11.1) |
| Morning goods - yeast raised | 50 | 330 | 1,038 | 48 (96.0) |
| Breakfast cereals * | **4** | **360** | **68** | **0 (0.0)** |
| Breakfast cereals | 4 | 360 | 68 | 0 (0.0) |
| Burgers in bun | **159** | **1,111** | **786** | **42 (26.4)** |
| All other burgers | 33 | 946 | 497 | 7 (21.2) |
| Burgers with cured meats | 110 | 1,229 | 906 | 30 (27.3) |
| Small burgers without cheese or other  cured meats | 16 | 639 | 554 | 5 (31.3) |
| Cakes, pastries, fruit pies and other pastry- based desserts * | **117** | **249** | **368** | **80 (68.4)** |
| Cakes | 103 | 265 | 368 | 68 (66.0) |
| Pastries | 2 | 180 | 377 | 2 (100.0) |
| Sweet pies and other shortcrust or choux  pastry based desserts | 12 | 120 | 366 | 10 (83.3) |
| Cheese * | **3** | **773** | **636** | **1 (33.3)** |
| Cheddar and other similar “hard pressed”  cheeses | 2 | 760 | 704 | 1 (50.0) |
| Processed cheese | 1 | 800 | 500 | 0 (0.0) |
| Chips and snacks * | **18** | **650** | **298** | **2 (11.1)** |
| Extruded and sheeted snacks | 7 | 760 | 276 | 0 (0.0) |
| Flavoured nuts | 1 | 480 | 108 | 0 (0.0) |
| Salt and vinegar products | 1 | 900 | 527 | 0 (0.0) |
| Savoury popcorn | 2 | 575 | 615 | 1 (50.0) |
| Standard potato chips | 7 | 550 | 225 | 1 (14.3) |
| Meat products * | **16** | **441** | **1,047** | **5 (31.3)** |
| Cooked uncured meat | 9 | 270 | 819 | 2 (22.2) |
| Frankfurters, hotdogs, and burgers | 6 | 700 | 634 | 2 (33.3) |
| Meat pie | 1 | 430 | 5575 | 1 (100.0) |
| Other processed potatoes * | **17** | **154** | **319** | **13 (76.5)** |
| Dehydrated instant mashed potato, as  consumed | 9 | 60 | 322 | 9 (100.0) |
| Other processed potato products | 8 | 260 | 315 | 4 (50.0) |
| Pasta meal | **87** | **1,157** | **910** | **37 (42.5)** |
| All other pasta dishes | 82 | 1,167 | 886 | 35 (42.7) |
| Lasagna, risotto, gnocchi and pasta with  cured meat additions | 5 | 993 | 1,297 | 2 (40.0) |
| Pizza | **606** | **445** | **712** | **383 (63.2)** |
| Take away style pizza with all other  toppings (per slice) | 288 | 343 | 589 | 202 (70.1) |
| Take away style pizza with cured meat  toppings (per slice) | 310 | 483 | 793 | 176 (56.8) |
| Traditional Italian style pizza with all other  toppings (per pizza) | 4 | 2,053 | 1929 | 3 (75.0) |
| Traditional Italian style pizza with cured  meat toppings (per pizza) | 4 | 3225 | 2146 | 2 (50.0) |
| Potato products | **63** | **315** | **532** | **44 (69.8)** |
| Seasoned chips and other potato products | 19 | 474 | 411 | 8 (42.1) |
| Seasoned fries | 44 | 247 | 584 | 36 (81.8) |
| Processed puddings * | **11** | **119** | **199** | **8 (72.7)** |
| All other processed puddings | 4 | 100 | 199 | 4 (100.0) |
| Cheesecake | 7 | 130 | 200 | 4 (57.1) |
| Ready meals * | **115** | **410** | **838** | **75 (65.2)** |
| Ready meal sides and accompaniments | 54 | 429 | 673 | 31 (57.4) |
| Ready meals | 61 | 392 | 984 | 44 (72.1) |
| Rice * | **6** | **173** | **332** | **4 (66.7)** |
| Flavoured rice | 4 | 230 | 434 | 3 (75.0) |
| Rice (unflavoured) | 2 | 60 | 129 | 1 (50.0) |
| Sandwiches | **300** | **876** | **818** | **159 (53.0)** |
| All other sandwiches | 193 | 709 | 762 | 116 (60.1) |
| Cured meat sandwiches | 107 | 1,176 | 918 | 43 (40.2) |
| Soups * | **47** | **235** | **509** | **45 (95.7)** |
| Soups | 47 | 235 | 509 | 45 (95.7) |
| Total | **1,914** | **596** | **712** | **1,083 (56.6)** |

*n=*1,914. Values are *n* (%), organized in alphabetical order according to food categories that exceed the UK National Salt Reduction Initiative (NSRI) targets [19]. Data is categorized by UK NSRI food category and subcategory. * UK NSRI food category for prepackaged foods was used because no category for restaurant foods was set.
